# Characterization of the CRM Gene Family and Elucidating the Function of OsCFM2 in Rice

**DOI:** 10.3390/biom10020327

**Published:** 2020-02-18

**Authors:** Qiang Zhang, Lan Shen, Deyong Ren, Jiang Hu, Li Zhu, Zhenyu Gao, Guangheng Zhang, Longbiao Guo, Dali Zeng, Qian Qian

**Affiliations:** State Key Laboratory of Rice Biology/China National Rice Research Institute, Chinese Academy of Agricultural Sciences, Hangzhou 310006, China; zhangqiang9024@126.com (Q.Z.); shenlan@caas.cn (L.S.); rendeyong@caas.cn (D.R.); hujiang588@163.com (J.H.); zhuli05@caas.cn (L.Z.); gaozhenyu@caas.cn (Z.G.); zhangguangheng@126.com (G.Z.); guolongbiao@caas.cn (L.G.); dalizeng@126.com (D.Z.)

**Keywords:** CRM domain, intron splicing, chloroplast development, rice, abiotic stress

## Abstract

The chloroplast RNA splicing and ribosome maturation (CRM) domain-containing proteins regulate the expression of chloroplast or mitochondrial genes that influence plant growth and development. Although 14 CRM domain proteins have previously been identified in rice, there are few studies of these gene expression patterns in various tissues and under abiotic stress. In our study, we found that 14 CRM domain-containing proteins have a conservative motif1. Under salt stress, the expression levels of 14 CRM genes were downregulated. However, under drought and cold stress, the expression level of some CRM genes was increased. The analysis of gene expression patterns showed that 14 CRM genes were expressed in all tissues but especially highly expressed in leaves. In addition, we analyzed the functions of OsCFM2 and found that this protein influences chloroplast development by regulating the splicing of a group I and five group II introns. Our study provides information for the function analysis of CRM domain-containing proteins in rice.

## 1. Introduction

The chloroplast RNA splicing and ribosome maturation (CRM) domain proteins play an important role in plant growth and development by influencing RNA-binding activity according to the characteristics and structure of their CRM domains [1,2]. CRM domain proteins regulate chloroplast- or mitochondria-related gene expression through post-transcriptional regulation [3,4]. It is reported that CRM proteins participate in the process of intron splicing and affect rRNA processing in chloroplasts [5,6,7]. In plants, mutations in CRM genes lead to an albino seedling phenotype, developmental delay, or sensitivity to abiotic stress [8,9,10,11].

Based on their structure and the count of CRM domains they contain, the CRM family proteins can be divided into four subfamilies: The CRS1 subfamily, CAF subfamily, subfamily 3, and subfamily 4 [1,5]. Among these, CRS1 subfamily, CAF subfamily, and subfamily 3 proteins have been reported to regulate the splicing of group I and group II introns [5]. Meanwhile, subfamily 4 proteins are associated with the assemblage of the large ribosomal subunit [10]. The functions of CRM domain proteins in *Arabidopsis thaliana* and maize have been widely reported. The first CRM gene, *ZmCRS1*, has been identified and cloned in maize; the ZmCRS1 protein contains three CRM domains that can directly bind to, and regulate splicing of *atpF* intron in chloroplasts [11]. The CFM3 protein also has three CRM domains, which are related to splicing of *trnG*, *rps16*, *ndhB*, *rpl16*, *petB*, and *petD* introns in *A. thaliana* [12]. CFM2 contains four CRM domains and affects group I and group II introns’ splicing in *A. thaliana* and maize [8,13]. Previous studies have indicated that CAF1 and CAF2 belong to the CAF subfamily, and can interact with CRS2 to form CAF1–CRS2 and CAF2–CRS2 complexes, which regulate the splicing of nine group II introns in chloroplasts [14,15]. Additionally, CRM domain proteins are involved in the splicing of mitochondrial gene introns. In *A. thaliana*, CFM9 belongs to subfamily 3, contains one CRM domain, and influences *nad1*, *nad2*, *nad4*, *nad5*, *nad7*, *rps3*, and *cox2* intron splicing in mitochondria [9]. Both AtmCSF1 and AtmCSF2 contain two CRM domains and are localized in the mitochondria. The AtmCSF1 affects *cox2*, *nad1*, *nad2*, *nad5*, *nad7*, and *rps3* intron splicing in mitochondria [4]. AtCFM4, in subfamily 4, contains one CRM domain and localizes to the chloroplast while it affects the assemblage of 16S and the 4.5S process. It has been reported that *atcfm4* mutants show sensitivity to abiotic stress, including cold and salt stress [10].

Previous studies have identified 14 CRM domain-containing proteins in rice [1,3]. Some of these CRM domain-containing proteins were found to be associated with intron splicing in chloroplast. Phenotypic analysis has shown that *al2*, *oscfm3*, and *oscaf1* mutants exhibit seedlings’ albino phenotype [12,16,17]. OsCRS1 has been reported to participate in the splicing of group I and group II introns, including *trnL*, *petD*, *ndhA*, *ndhB*, and *ycf3-1* [16]. Meanwhile, OsCFM3 participates in the splicing of group II introns involving *rps16*, *rpl16*, *ndhB*, and *petD* [12]. Our previous studies showed that OsCAF1 interacts with the C-terminal of OsCRS2 to form OsCAF1–OsCRS2 complex that can influence introns’ splicing of *atpF*, *rpl2*, *rps12*, *ndhA*, *ndhB*, and *ycf3* [17]. Until now, the functions of most CRM domain proteins in rice have not been reported.

In the present study, we analyzed the genes and protein structures of 14 CRM domain proteins and identified the expression patterns of these genes in various tissues and under abiotic stress. We edited *Os04g0464800* (*OsCFM2*) using a CRISPR/Cas9 gene editing system and obtained two allele mutants, *oscfm2-1* and *oscfm2-2*, which both exhibited an albino seedling phenotype. We found that OsCFM2 influences group I and group II intron splicing in chloroplast and plays an important role in chloroplast development in rice.

## 2. Materials and Methods 

### 2.1. Sequence Analysis and Phylogenetic Tree Construction

We acquired 14 CRM protein sequences and gene sequences from the rice genome annotation project database. The gene structures of 14 CRM genes’ family were analyzed by the online gene structure display server (GSDS, http://gsds.cbi.pku.edu.cn/) [18]. The conserved motifs of 14 CRM domain proteins were analyzed using online motif elicitation software (MEME, http://meme-suite.org/) [19]. The parameters were as follows: 10 motifs in maximum, motif width with 6–50 residues, and E-values < 1.00 × 10^−20^. A phylogenetic tree was built using the neighbor-joining method by 1000 bootstrap in MEGA-X software [20]. 

### 2.2. Plant Materials and Abiotic Stress Treatments

To study the expression pattern of CRM genes in various tissues, we obtained roots (R), culm (C), young leaves (YL), expansion leaves (EL), and young spikelet (YS) at the booting stage. Germinated seeds (Nipponbare) were transferred to a nutrient solution in an incubator and kept at 30 °C under a 16 h light/8 h dark cycle. Accordance to previous studies, we treated rice seedlings at the three-leaf stage with low temperature (15 °C), salt stress (100 mM NaCl), and drought stress (10% PEG) for 3 days [21,22,23]. After taking leaves, samples were stored at −80 °C for further analysis. 

OsCFM2/*oscfm2-1* and OsCFM2/*oscfm2-2* heterozygous plants were obtained using a CRISPR/Cas9 gene editing system with the Nipponbare, which were used as wildtype (WT). The *oscfm2-1* and *oscfm2-2* mutants were acquired from the T_1_ generation of OsCFM2/*oscfm2-1* and OsCFM2/*oscfm2-2* heterozygous plants, respectively. The WT and two allele mutants were grown in a greenhouse under a 16 h light/8 h dark cycle at a constant temperature of 30 °C.

### 2.3. OsCFM2 Knockout with a CRISPR/Cas9 System

The rice gene editing methods were dependent on the CRISPR/Cas9 system [24]. For the first exon of *OsCFM2*, we designed one gRNA target sequence (GCTCCTCCTCTTCCTCCCCCA) to construct an intermediate vector, SK-gRNA-gOsCFM2-g1. Using Kpn I and Bgl II, intermediate vectors were digested, and then gRNA-gOsCFM2-g1 was assembled into the pC1300-Cas9 vector. 

### 2.4. Measurement of Chlorophyll Content and Fv/Fm

Fromthe 18-day seedlings, we took 50-mg leaf samples, cut them into small pieces, and immersed them in 20 mL of 95% ethanol for 48 h in darkness at 4 °C. We then used a UV-1800PC spectrophotometer to measure the absorbance of the samples at 665, 649, and 470 nm. Then, we calculated the chlorophyll a, chlorophyll b, and total chlorophyll content according to the method of Lichtenthaler [25].

For Fv/Fm measurement, the WT and two allele mutants in the18-day seedlings were kept in darkness for 30 min. Then, we measured the Fv/Fm with a handheld fluorometer according to the manufacturer’s instructions. 

### 2.5. Transmission Electron Microscopy (TEM)

Transmission electron microscopy analysis was carried out as previously described [26]. In the two-leaf stage, the leaves of WT plants and *oscfm2-1* mutants were collected and cut into 0.1 cm × 0.1 cm pieces. Leaf samples were fixed in 2.5% glutaraldehyde and 1% OsO4. Then, samples were dehydrated via an ethanol series and embedded in resin. Samples were stained with uranyl acetate and alkaline lead citrate and after observed using a HitachiH-7500 transmission electron microscope.

### 2.6. RNA Extraction and Quantitative Real-Time PCR

Total rice RNA was extracted from samples using an RNA Extraction Kit (TaKaRa) according to the manufacturer’s instructions. First-strand cDNA was obtained using a ReverTra Ace qPCR RT Kit (TOYOBO). The quantitative real-time PCR (qRT-PCR) experimental procedure was as described in a previous study [27]. In the present study, the *OsAction1* gene was used as an internal control, and the relative expression levels of genes were calculated using the 2^−ΔΔCT^ method [28]. All qRT-PCR primers used are listed in Table S1.

### 2.7. Chloroplast Gene Intron Splicing Analysis

To analyze intron splicing in chloroplast genes, chloroplast genes containing at least one intron were selected, and amplified using the RT-PCR method. The RT-PCR procedure was as follows: 96 °C for 5 min, followed by 30 cycles of 96 °C for 40 s, 60 °C for 30 s, 72 °C for 45 s, and a final elongation step at 72 °C for 10 min. All RT-PCR primers used in the present study are listed in Table S1.

## 3. Results

### 3.1. Gene and Protein Structure Analysis of Rice CRM Domain Proteins

**Figure 1:**
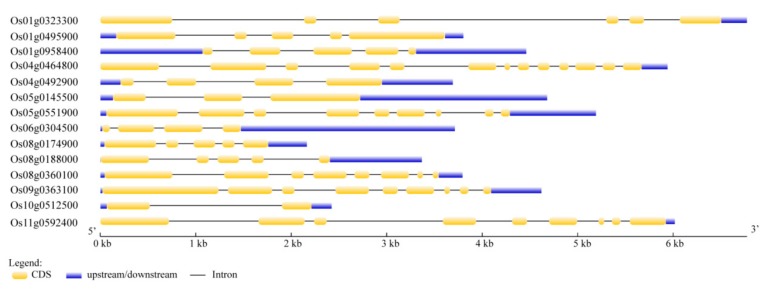
Structural analysis of CRM genes’ family. Yellow and blue boxes indicate coding sequences and upstream or downstream untranslated regions, respectively. Black lines indicate introns. The scale bar at the bottom shows the length of the CRM genes in Kb.

**Figure 2:**
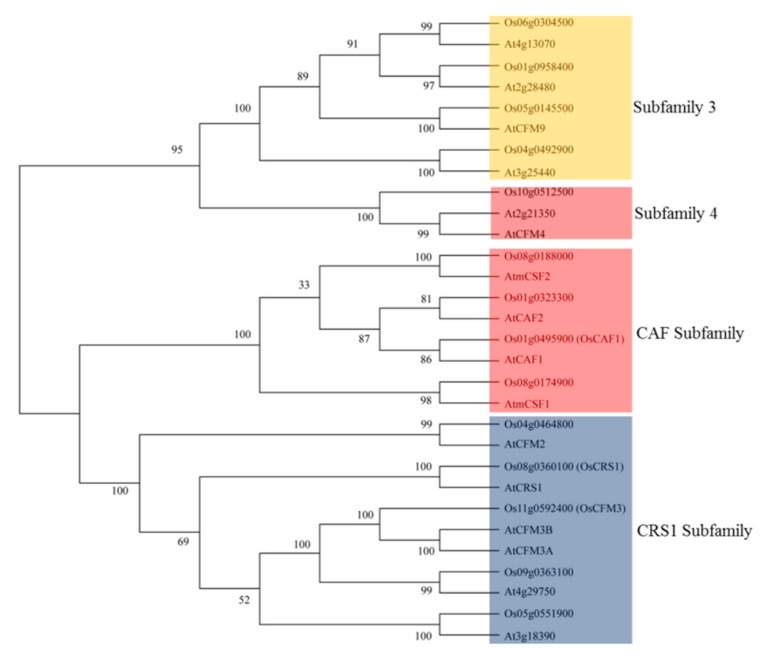
Phylogenetic tree of CRM domain proteins in rice and *A. thaliana*. The full amino acid sequences of the CRM domain proteins were aligned by MUSCLE and the neighbor-joining tree was constructed in MEGA X.

The 14 CRM genes of rice are distributed among eight chromosomes: 1, 4, 5, 6, 8, 9, 10, and 11 chromosomes. Gene structure analysis indicated that the CRM genes contained 2–11 exons and the coding sequences of these genes ranged from 759 to 3039 base pairs (bp) in length (Figure 1). The CRM domain proteins encoded by these genes contained 252–1012 amino acids and their molecular weights were in the range of 27.75–111.29 kDa (Table S2). We obtained *Arabidopsis* CRM domain protein sequences from the *Arabidopsis* information resource database to build a phylogenetic tree (Figure 2). All proteins in the CRS1 subfamily except OsCFM2 have four CRM domains, and OsCRS1, OsCFM3, Os05g0551900, and Os09g0363100 contained three CRM domains. In the CAF subfamily, all proteins contained two CRM domains while in subfamily 3 and subfamily 4, all proteins contained only one CRM domain (Table S2). Only one CRM domain protein was identified in subfamily 4. Through TargetP program prediction, it was determined that nine of these CRM domain proteins were located in the chloroplast, and five CRM domain proteins located in the mitochondrion (Table S2). Those proteins’ actual subcellular localization needed to be verified by an experiment.

**Figure 3:**
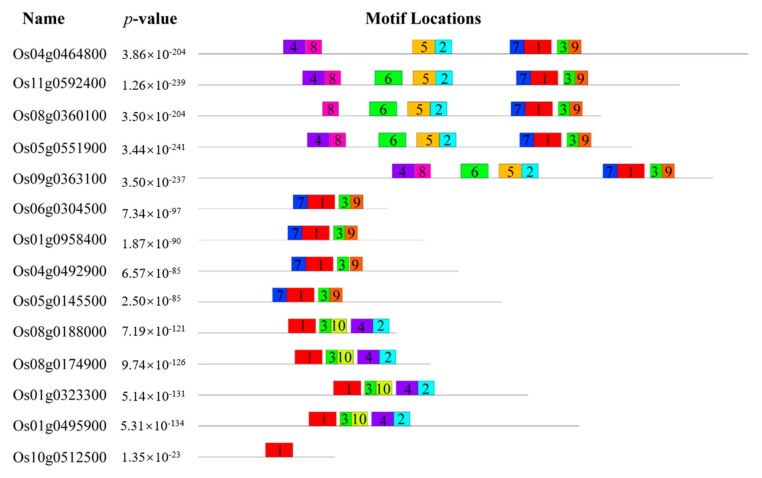
Conserved putative motif analysis of CRM domain proteins in rice. Ten putative motifs are shown in different colored boxes.

In addition, to further understand the structures of these 14 CRM domain proteins, we predicted their conserved putative motifs using the online program MEME. In total, 10 motifs were identified and named motif 1 to motif 10 (Figure S1). We found that 14 CRM domain proteins contained conserved putative motif 1. It is interesting to note that motif 1 was located in the C-terminal of the CRS1 subfamily proteins. Motif 5 and motif 8 only existed in the CRS1 subfamily in rice. Only CAF subfamily proteins contain motif10 (Figure 3).

### 3.2. Expression Analysis of CRM Genes in Various Tissues

**Figure 4:**
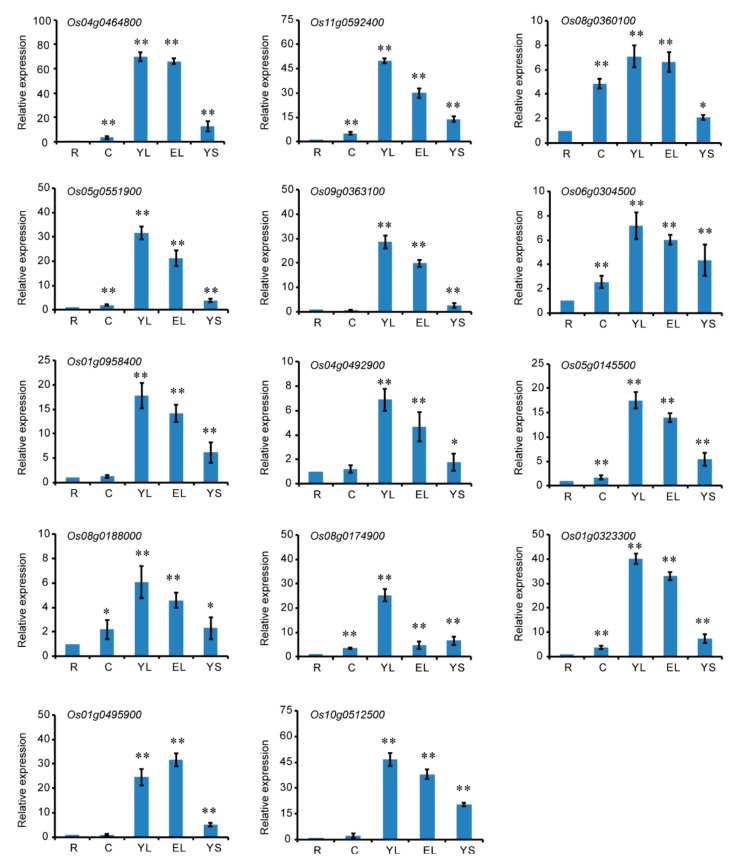
Analysis of CRM gene expression in various rice tissues by qRT-PCR. Tissues, including root (R), culm (C), young leaf, expansion leaf (EL), and young spikelet (YS), at the booting stage. The relative expression level in each tissue was controlled by the gene expression level in the root. Data are the mean ± SD for three biological replicates. * *p* < 0.05, ** *p* < 0.01 by Student’s t-test.

In order to study the expression patterns of CRM family genes in various tissues, we sampled roots (R), culm (C), young leaves (YL), expanded leaves (EL), and young spikelet (YS) in the booting stage for qRT-PCR analysis. The results indicated that the CRM family genes were expressed in all tissues, but particularly high expressed in green tissues, especially in YL and EL, suggesting that they might function in leaf development in rice (Figure 4). Some of the genes have previously been reported to be involved in chloroplast development, such as *OsCAF1* and *OsCRS1*.

### 3.3. Expression Analysis of CRM Genes under Abiotic Stress

**Figure 5:**
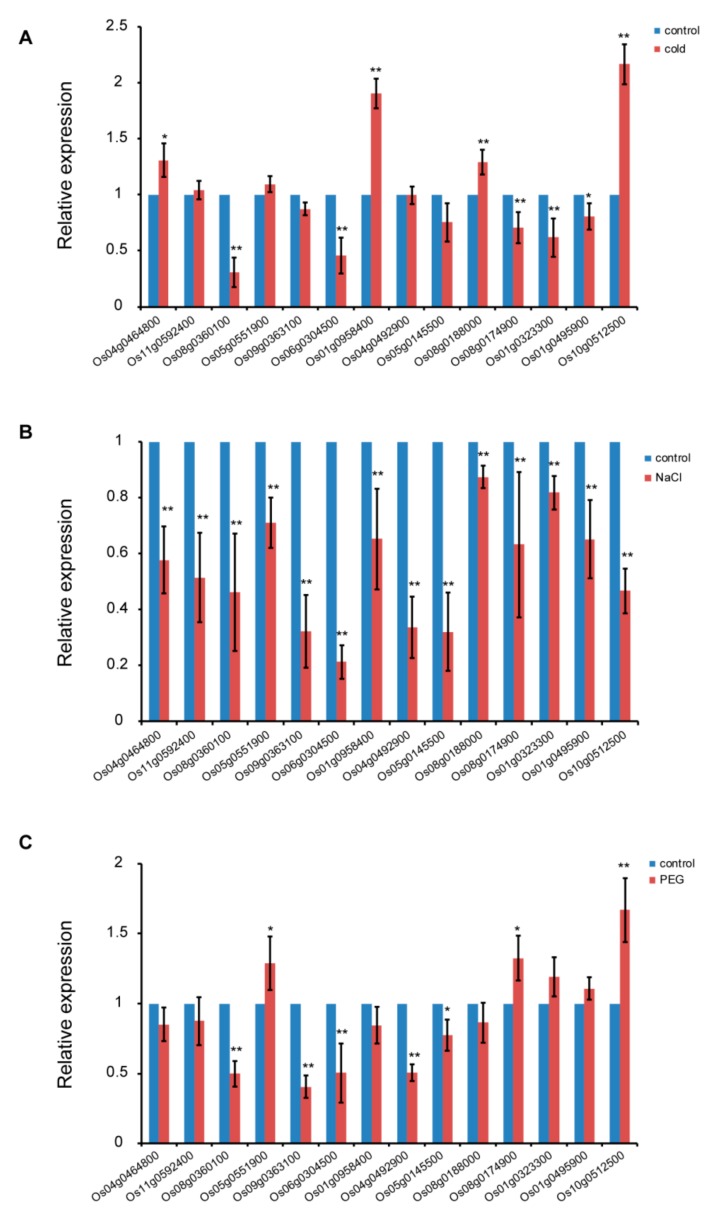
Analysis of CRM gene expression in the leaves of three-leaf stages under (**A**) cold stress (15 °C), (**B**) salt stress (100mM NaCl), and (**C**) drought stress (10% PEG). Data are the mean ± SD for three biological replicates. * *p* < 0.05, ** *p* < 0.01 by Student’s t-test.

Research has shown that CRM domain proteins play an important role in plant development and growth [9,10,17]. Additionally, the CRM genes could also be involved in response to abiotic stress [29]. In *A. thaliana*, AtCFM4 belongs to subfamily 4, shown to be sensitive to salt and cold stresses [10]. However, the effect of abiotic stress on CRM gene expression in rice has not yet been reported. Abiotic stresses include cold temperatures, high salt, and drought, which affect rice growth. To investigate whether the expression of CRM genes was affected by abiotic stress, the expression levels of 14 CRM genes were evaluated by qRT-PCR analysis under abiotic stress. Under cold stress, the expression levels of five and four CRM genes were dramatically downregulated and upregulated, respectively, while five genes involving *Os11g0592400*, *Os05g0551900*, *Os09g0363100*, *Os04g0492900*, and *Os05g0145500* expression levels showed no significant change (Figure 5A). Under salt stress, the expression levels of 14 CRM genes were significantly downregulated (Figure 5B). Under drought stress, the expression levels of five and three CRM genes were significantly downregulated and upregulated, respectively, and the expression levels of six genes, including *Os04g0464800*, *Os11g0592400*, *Os01g0958400*, *Os08g0188000*, *Os01g0323300*, and *Os01g0495900* expression levels, showed no significant change (Figure 5C).

### 3.4. OsCFM2 (Os04g0464800) Influences Chloroplast Development in Rice

**Figure 6:**
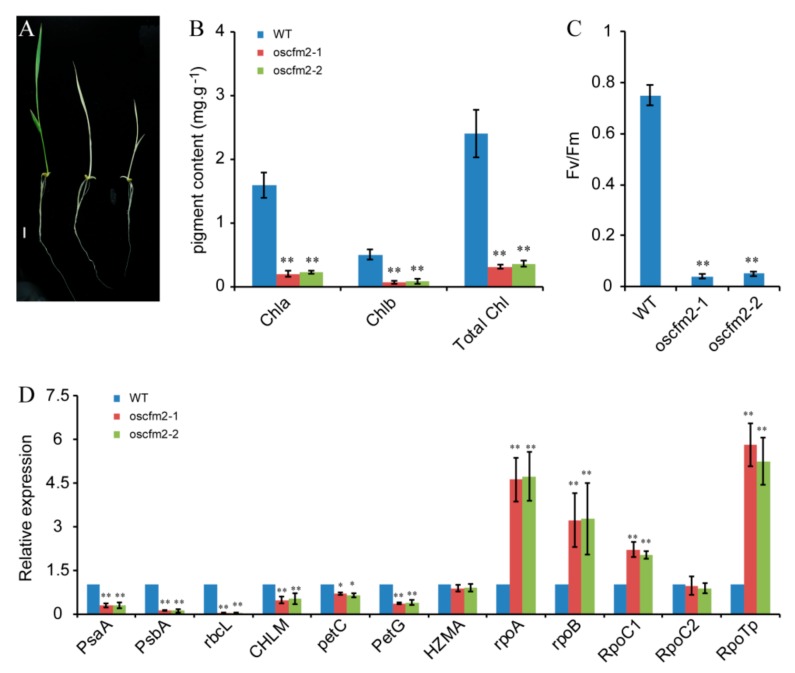
Mutation of *OsCFM2* causes an albino phenotype in rice leaves. (**A**) Phenotypes of the WT (left) and *oscfm2-1* (middle) and *oscfm2-2* mutants (right) at the seedling stage. Scale bar = 0.5 cm. (**B**) Chlorophyll contents of WT and *oscfm2-1* and *oscfm2-2* mutants at the seeding stage. (**C**) The Fv/Fm value of the WT and *oscfm2-1* and *oscfm2-2* mutants at the seeding stage. (**D**) qRT-PCR analysis of genes related to chloroplast synthesis and photosynthesis in the WT and *oscfm2-1* and *oscfm2-2* mutants. Values represent the mean ± SD (n = 3). ** *p* < 0.01 by Student’s t-test.

The *Os04g0464800* gene belongs to the CRS1 subfamily and was homologous with *AtCFM2*, so we named this gene *OsCFM2*. The function of OsCFM2 has not been studied until now. To study the function of OsCFM2, we constructed the pC1300-Ubi::Cas9-gOsCFM2^-target1^ vector and transformed the vector into Nipponbare using an *Agrobacterium*-mediated method [30]. In the T_0_ generation, we obtained nine transgenic plants. The sequencing results showed that only two of these plants, OsCFM2/*oscfm2-1* and OsCFM2/*oscfm2-2*, were heterozygotes plants. In the T_1_ generation, we obtained two allele mutants: *oscfm2-1* and *oscfm2-2*, from OsCFM2/*oscfm2-1* and OsCFM2/*oscfm2-2* plants, respectively. Phenotypic analysis indicated that the mutants showed albinism in the seedling stage (Figure 6A). In *oscfm2-1* and *oscfm2-2*, the deletion of 2 and 11 bp in the first exon of the *OsCFM2* gene coding sequence region, respectively, led to frame-shift mutations that resulted in a premature stop codon (Figure S2). These results suggest that the mutation of *OsCFM2* leads to the albino seedling phenotype.

The chlorophyll a, chlorophyll b, and total chlorophyll contents were markedly reduced in *oscfm2-1* and *oscfm2-2* mutants compared to the WT (Figure 6B). Additionally, the Fv/Fm values were significantly lower in the *oscfm2-1* and *oscfm2-2* mutants than in the WT (Figure 6C). We also analyzed the expression of genes related to chloroplast development and photosynthesis in *oscfm2-1* and *oscfm2-2* mutants and the WT. The results showed that the relative expression levels of genes associated with chloroplast development and photosynthesis were significantly changed in mutants compared to the WT. For example, the expression levels of *PsaA*, *PsbA*, *rbcL*, *petC*, *PetG*, and *CHLM* were remarkably reduced in *oscfm2-1* and *oscfm2-2* mutants (Figure 6D), whereas genes encoding NEP and PEP, such as *rpoA*, *rpoB*, *RpoC1*, and *RpoTp*, were significantly increased (Figure 6D).

**Figure 7:**
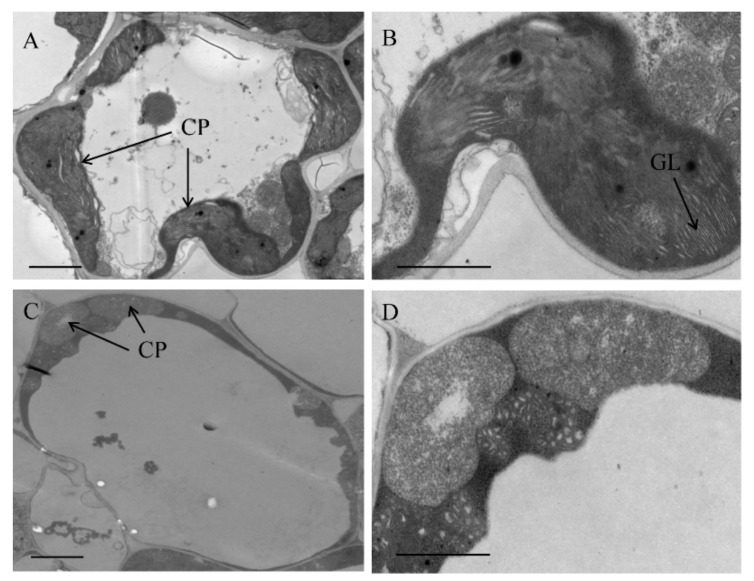
Chloroplast ultrastructure of WT (**A**, **B**) and *oscfm2-1* mutants (**C**, **D**). cp, chloroplast, gl, grana lamella. Bars = 2.0 μm.

We observed the ultrastructure of chloroplasts in 18-day-old WT and *oscfm2-1* mutant leaves by TEM. Normal chloroplasts and grana stacks were found in the WT (Figure 7A,B). In contrast, in *oscfm2-1* mutant, the chloroplast was abnormal, and appeared to stop developing at the proplastid stage (Figure 7C,D). These observations suggest that a mutation in *OsCFM2* influences chloroplast development. Taken together, our results suggest that OsCFM2’s lack of function leads to abnormal chloroplast development in rice.

### 3.5. OsCFM2 Influences the Splicing of Group I and Group II Introns

To test whether OsCFM2 affected the splicing of chloroplast gene introns, group I and group II introns were amplified from the two allele mutants and WT. The results indicated that the splicing of five group II introns, including *rpl2*, *rps12*, *ycf3-1*, *atpF*, and *ndhA*, were affected in mutants compared to the WT (Figure 7A). Unlike in the WT, the *atpF* and *ndhA* introns were not completely spliced in *oscfm2-1* and *oscfm2-2* mutants. The *trnL* group I intron was also not spliced in mutants (Figure 7A). The qRT-PCR analysis showed that *rpl2*, *rps12*, *ycf3*, *atpF*, *ndhA*, and *trnL* gene expression levels were decreased in *oscfm2-1* and *oscfm2-2* mutants compared with the WT. These results suggest that OsCFM2 may affect chloroplast development by influencing the splicing of group I and five group II introns. However, the other 10 group II introns had normal splicing in *oscfm2-1* and *oscfm2-2* mutants compared with the WT (Figure S3).

## 4. Discussion

Genomic structural analysis indicated that 14 CRM genes in rice contain introns (Figure 1). In rice, 5, 4, 4, and 1 of the 14 CRM proteins are classified as CRS1, CAF, subfamily 3, and subfamily 4 proteins, respectively (Figure 2). Protein motif analysis showed that the protein motifs in each subfamily were highly conserved (Figure 3). Proteins in the CRS1 subfamily were found to contain more motifs than those in other subfamilies. All proteins in the CRS1 subfamily except OsCRS1 contained eight motifs while the other four proteins contained nine motifs. There were five and four motifs in the CAF subfamily and subfamily 3, respectively (Figure 3). Our results indicated that the proteins in each subfamily harbored different numbers of conserved motifs, suggesting that these proteins might have different functions in rice. For example, in the CRS1 subfamily, OsCRS1 influences intron splicing in *trnL* and multiple group II introns [16]. Meanwhile, in the CAF subfamily, OsCAF1 regulates the splicing of some group II introns in chloroplasts [17]. The functions of the CRM domain proteins of different subfamilies may therefore be differentiated.

Expression analysis showed that the 14 CRM genes were most highly expressed in rice leaves (Figure 4); this result was consistent with previous studies. It is reported that *OsCRS1* and *OsCAF1* are highly expressed in rice leaves [16,17]. Phenotypic analysis showed that *oscrs1* and *oscaf1* mutants exhibit an albino phenotype in the seedling stage [16,17]. This result suggested that the CRM family genes might play a key role in chloroplast development in leaves. We noticed that the CRM family genes were also highly expressed in young spikelets, suggesting that they might function in spikelet development (Figure 4). It iss reported that ZmmCSF1 and AtmCSF1 were essential for seed development [4,31]. Therefore, *Os08g0174900*, which is homologous with *AtmCSF1*, may also play an important role in seed development in rice.

It has been reported that in *A. thaliana*, AtCFM4 and AtCFM9 influence seedling growth under cold and salt stress [9,10]. In the present study, we found that the expression levels of CRM genes were changed under abiotic stress, especially under salt stress, which might affect intron splicing in chloroplast or mitochondrial genes, leading to increased sensitivity to abiotic stress in rice (Figure 5). Further study is required to elucidate the molecular mechanism of the CRM domain protein response to abiotic stress.

**Figure 8:**
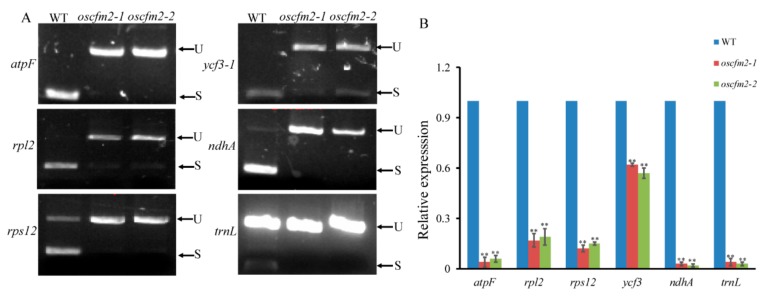
Intron splicing and gene expression analysis. (**A**) Splicing analysis of chloroplast genes introns in WT and *oscfm2-1* and *oscfm2-2* mutants. S indicates spliced transcripts. U indicates unspliced transcripts. (**B**) Expression analyzed of *atpF*, *rpl2*, *rps12*, *ycf3*, *ndhA*, and *trnL* in WT and mutants. Values represent the mean ± SD (n = 3). ** p < 0.01 by Student’s t-test.

The CRM domain proteins play key roles in plant growth and development [3,32]. Many intron splicing factors have been identified in different plants, involving PPR family and CRM family proteins [33,34]. For example, WSL4, SOT5, and THA8 affect chloroplast genes’ intron splicing in rice, *A. thaliana*, and maize, respectively [35,36,37]. There is evidence to indicate that CRM proteins have a high affinity for group II intron RNA in vitro [38]. In rice, some CRM proteins might have similar functions, influencing the splicing of group II introns. It has been reported that *OsCAF1*, *OsCFM3*, and *OsCRS1* could influence the splicing of multiple group II introns in rice [12,16,17]. The molecular mechanism underlying the regulation of chloroplast gene intron splicing by CRM proteins might be different in various plants. In *A. thaliana* and maize, AtCRS1 and ZmCRS1 only influence *atpF* intron splicing while OsCRS1 influences the splicing of many introns, including *atpF*, *petD*, *ndhA*, *ndhB*, *ycf3-1*, and *trnL* [11,13,16]. In the present study, we found that OsCFM2 affected intron splicing in *ycf3-1*, *rpl2*, *rps12*, *atpF*, *ndhA*, and *trnL*, which is unlike AtCFM2 in *A. thaliana* (Figure 8).

The *oscfm2-1* and *oscfm2-2* mutants exhibited an albino phenotype and survived for about three weeks (Figure 6A). In mutants, chloroplast development is abnormal and chlorophyll content was significantly decreased (Figure 6B). This result suggests that disruption of the function of OsCFM2 affected chloroplast development. In the rice genome, except for *OsCFM2*, the CRS1 subfamily contains *OsCRS1*, *OsCFM3*, *Os05g0551900*, and *Os09g0363100*. We examined the expression levels of these four genes among WT and mutants. The results showed that their expression levels were upregulated in *oscfm2-1* and *oscfm2-2* mutants compared to the WT (Figure S3). This result suggests that on the one hand, there might be negative regulation between *OsCFM2* and other CRS1 subfamily genes at the transcript level. On the other hand, this may be a feedback mechanism in which the cells try to increase the expression of the four CRS1 family’s genes to improve the splicing efficiency of the chloroplast genes’ intron but failed to compensate for the phenotype of the *OsCFM2* mutation.

The OsCAF1 influences intron splicing in *atpF*, *rpl2*, *rps12*, *ndhB*, *ycf3*, and *ndhA* [13]. OsCRS1 not only affected the splicing of the *trnL* intron but also the *atpF*, *rpl2*, *ndhA*, *ndhB*, *petB*, and *ycf3* introns in rice [17]. Therefore, OsCFM2 may have functional overlap with OsCAF1 and OsCRS1, but it is not redundant. These results indicate that OsCFM2 is functional and necessary for chloroplast development.

## 5. Conclusions

In the present study, we analyzed the expression patterns of 14 CRM genes’ family in different rice tissues and under abiotic stress. Moreover, we characterized the function of OsCFM2, which plays a key role in chloroplast development in rice. Our study provides information for the functional analysis of CRM domain proteins in rice.

## Supplementary Materials

The following are available online at https://www.mdpi.com/2218-273X/10/2/327/s1, Figure S1: MEME analysis shows each motif of the 14 CRM protein in rice, Figure S2: Mutation types of *oscfm2-1* and *oscfm2-2* mutants, Figure S3: Splicing analysis of chloroplast gene introns, Figure S4: Expression analysis of CRS1 subfamily genes in WT rice plants and *oscfm2-1* and *oscfm2-2* mutants, Table S1: Primers for this study, Table S2 Characteristics of 14 CRM domain proteins in rice.
