# Supplementary figures and images for: Characterization of the CRM Gene Family and Elucidating the Function of OsCFM2 in Rice
Source: Biomolecules. 2020 Feb 18;10(2):327. doi: 10.3390/biom10020327 (PMC7072668; doi:10.3390/biom10020327)

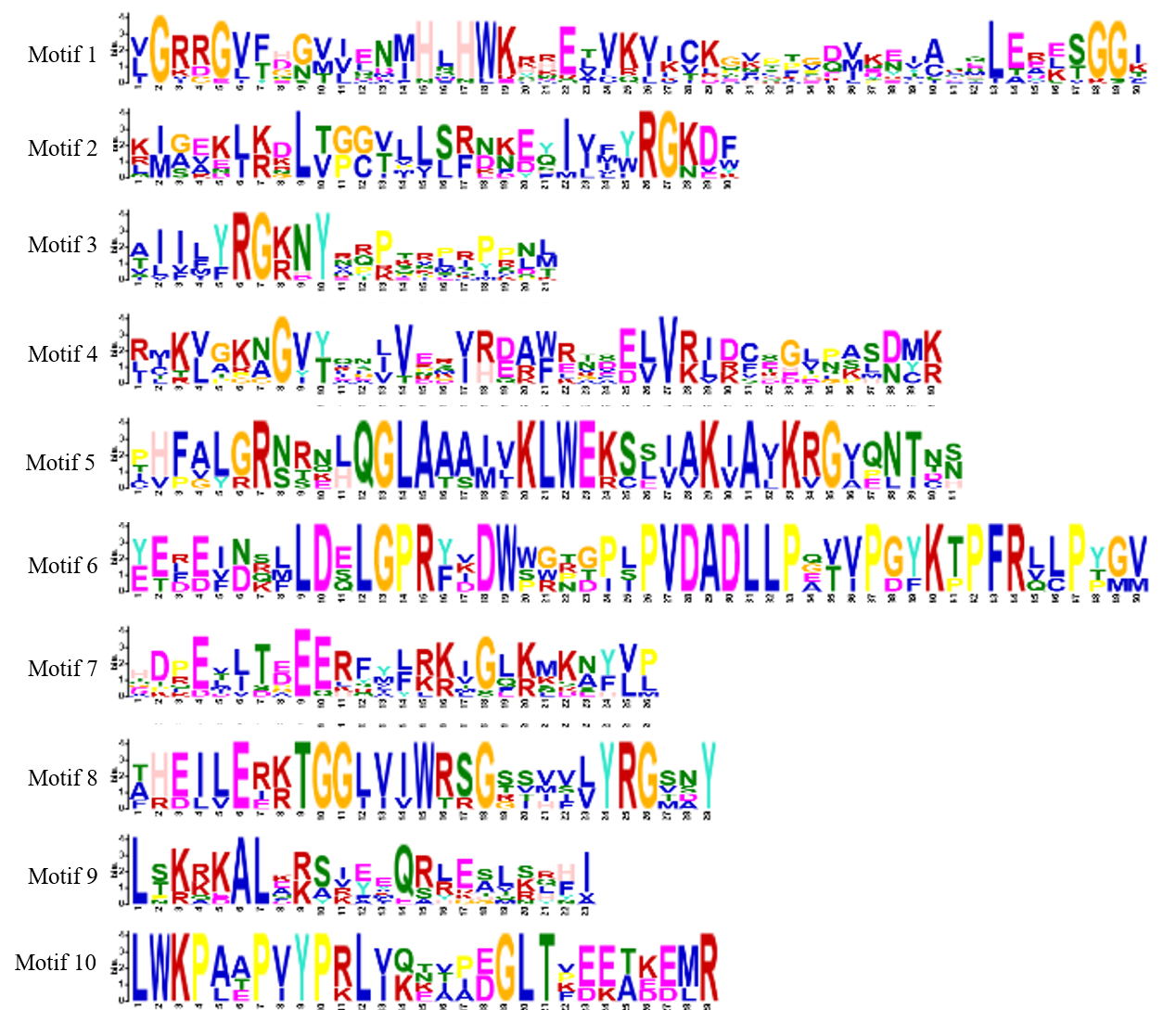

Supplement: Supplementary file 1 [file biomolecules-10-00327-s001.zip › Supplementary/Figure S1 MEME analysis shows each motif of the 14 CRM protein in rice.tif]

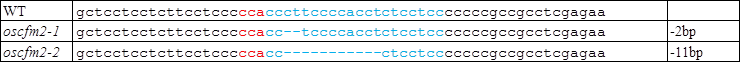

Supplement: Supplementary file 1 [file biomolecules-10-00327-s001.zip › Supplementary/Figure S2 Mutation types of oscfm2-1 and oscfm2-2 mutants..tif]

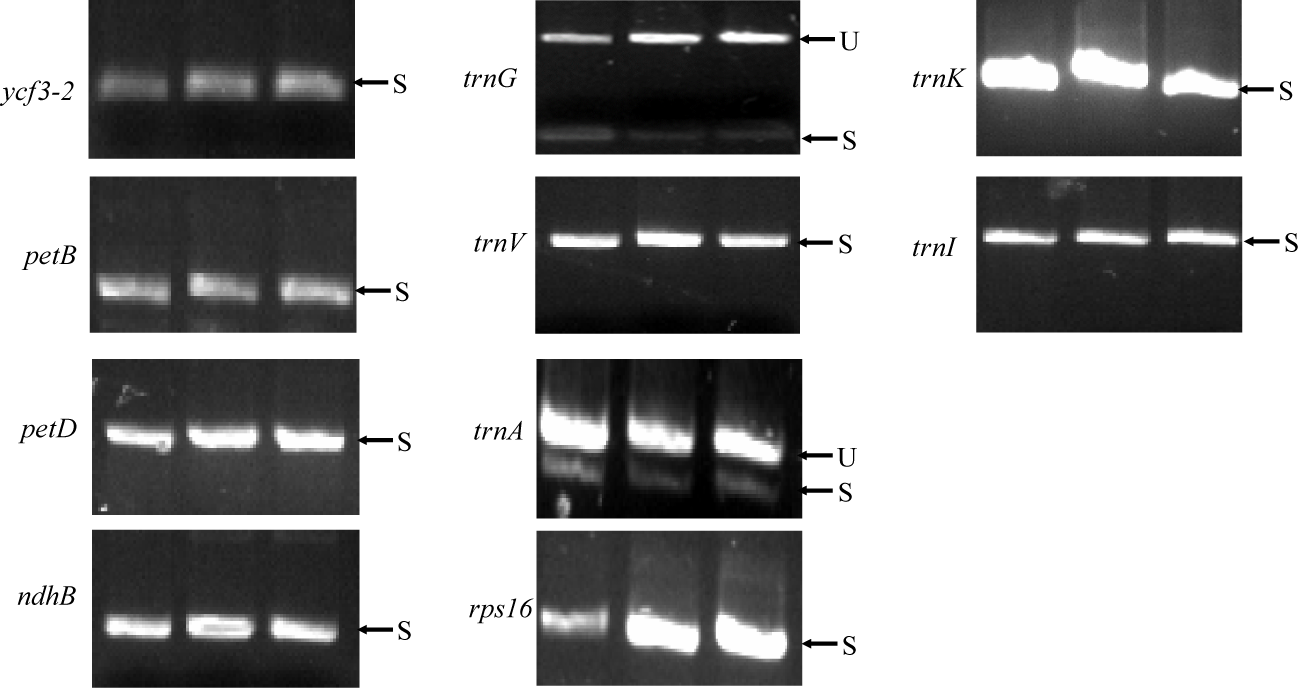

Supplement: Supplementary file 1 [file biomolecules-10-00327-s001.zip › Supplementary/Figure S3 Splicing analysis of chloroplast gene introns.tif]

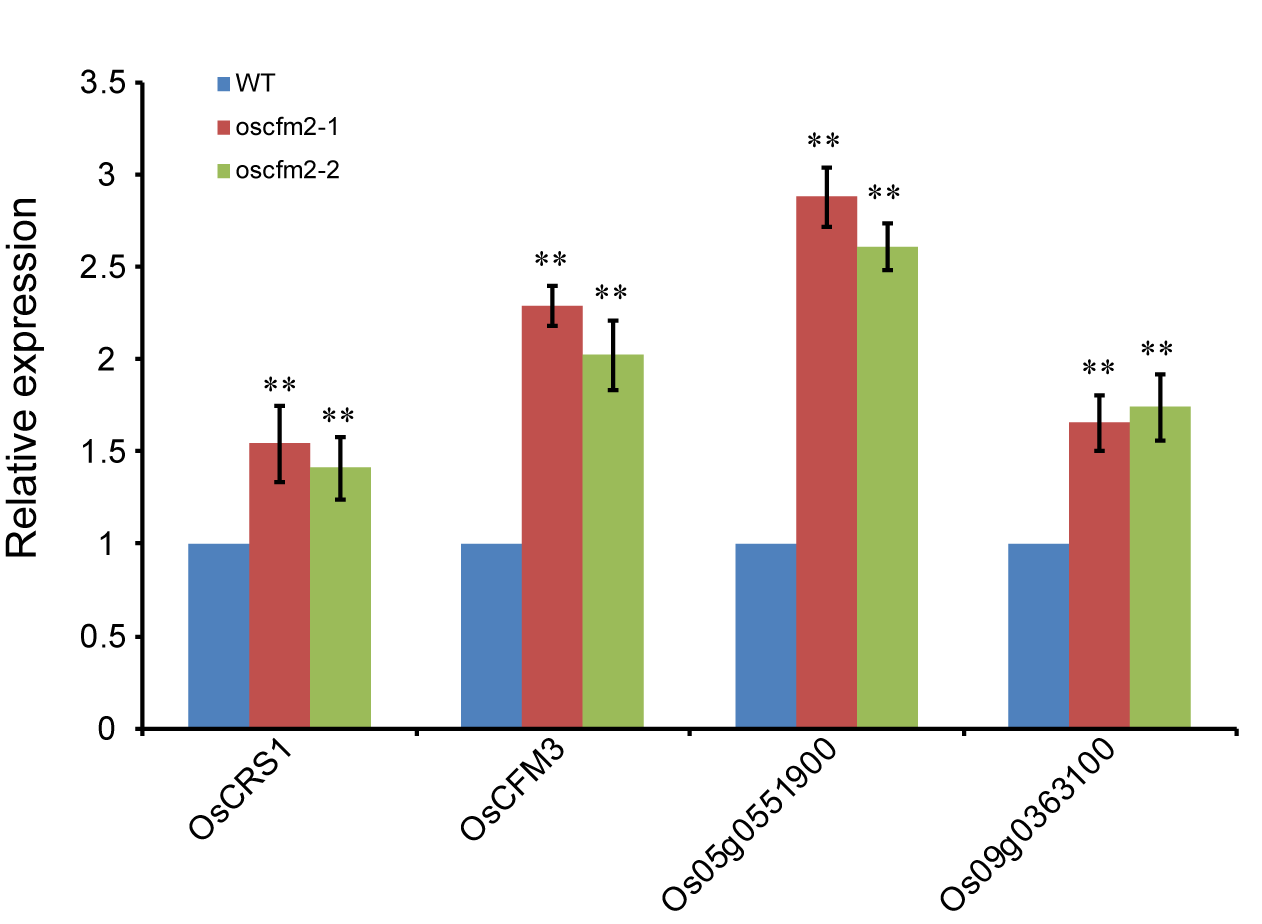

Supplement: Supplementary file 1 [file biomolecules-10-00327-s001.zip › Supplementary/Figure S4 Expression analysis of CRS1 subfamily genes in WT rice plants and oscfm2-1 and oscfm2-2 mutants.tif]
